# *Semla:* a versatile toolkit for spatially resolved transcriptomics analysis and visualization

**DOI:** 10.1093/bioinformatics/btad626

**Published:** 2023-10-16

**Authors:** Ludvig Larsson, Lovisa Franzén, Patrik L Ståhl, Joakim Lundeberg

**Affiliations:** Department of Gene Technology, KTH Royal Institute of Technology, Science for Life Laboratory, Tomtebodavägen 23, 171 65 Solna, Stockholm, Sweden; Department of Gene Technology, KTH Royal Institute of Technology, Science for Life Laboratory, Tomtebodavägen 23, 171 65 Solna, Stockholm, Sweden; Respiratory & Immunology, Neuroscience, Vaccines & Immune Therapies Safety, Clinical Pharmacology & Safety Sciences, BioPharmaceuticals R&D, AstraZeneca, Pepparedsleden 1, 431 83 Mölndal, Gothenburg, Sweden; Department of Gene Technology, KTH Royal Institute of Technology, Science for Life Laboratory, Tomtebodavägen 23, 171 65 Solna, Stockholm, Sweden; Department of Gene Technology, KTH Royal Institute of Technology, Science for Life Laboratory, Tomtebodavägen 23, 171 65 Solna, Stockholm, Sweden

## Abstract

**Summary:**

Spatially resolved transcriptomics technologies generate gene expression data with retained positional information from a tissue section, often accompanied by a corresponding histological image. Computational tools should make it effortless to incorporate spatial information into data analyses and present analysis results in their histological context. Here, we present *semla*, an R package for processing, analysis, and visualization of spatially resolved transcriptomics data generated by the Visium platform, that includes interactive web applications for data exploration and tissue annotation.

**Availability and implementation:**

The R package *semla* is available on GitHub (https://github.com/ludvigla/semla), under the MIT License, and deposited on Zenodo (https://doi.org/10.5281/zenodo.8321645). Documentation and tutorials with detailed descriptions of usage can be found at https://ludvigla.github.io/semla/.

## 1 Introduction

The rise of new transcriptomics technologies has enabled the exploration of genome-wide expression profiles in tissues. Spatially resolved transcriptomics (SRT) methods facilitate quantification of gene expression levels in tissue sections while preserving positional information. SRT thus allows researchers to analyze cellular and biological processes in the context of their tissue microenvironment. Several SRT technologies have emerged, offering different resolutions, sample sizes, sample throughput, transcriptome coverage, and sensitivity (Ståhl *et al.* 2016, Rodriques *et al.* 2019, Cho *et al.* 2021, Fu *et al.* 2022). In recent years, commercialization of SRT technologies has further improved their accessibility and ease of use, increasing the number of publicly available SRT datasets. With a constantly growing number of SRT datasets, there is an increasing need for accessible computational tools that facilitate efficient exploration, visualization, and analysis.

Visium, an SRT technology from 10x Genomics which to date is the most widely used SRT method (Moses *et al.* 2022), enables genome-wide transcriptome profiling of tissue sections with an area up to 11×11 mm^2^ (Visium Spatial Gene Expression, 10x Genomics). The spatial resolution is determined by the size of individual capture elements known as “spots,” each measuring 55 µm in diameter. A bright field image is taken of the tissue’s histological features, and by mapping gene expression levels onto the image, it adds morphological information that can be used to guide downstream analyses. Consequently, computational frameworks dealing with Visium data should facilitate seamless integration of the two data modalities. Several applications and libraries are available for analysis and exploration of Visium data (Stuart *et al.* 2019, Bergenstrahle *et al.* 2020, Dries *et al.* 2021, Palla *et al.* 2022, Pardo *et al.* 2022, Righelli *et al.* 2022), where the *Seurat* R package is one of the most popular. Seurat provides the infrastructure to handle single-cell transcriptomics and SRT data and includes a versatile toolbox of computational methods for analytical tasks. Owing to its popularity, several analysis methods have therefore been built to be compatible specifically with *Seurat* objects, broadening the scope of Seurat based analysis workflows. *Seurat* provides core functionalities for handling data from a variety of SRT platforms as of version 3.2 and their latest releases. However, the availability of effective interactive features and spatially aware analysis tools for Visium data are still limited. To bridge some of the current gaps, we provide a new R package that extends the *Seurat* toolbox for spatial analyses. Our package delivers extra Visium data processing tools for users with variable programming expertise, featuring well-documented and interactive components to simplify the analytical process.

## 2 Implementation and description

We have developed *semla*, a toolbox for data processing, exploration, analysis, and visualization of spatial gene expression patterns in tissues. *Semla* is written in the R programming language (≥v.4.1) and takes advantage of the *tidyverse* (Wickham *et al.* 2019) framework for data handling and the *patchwork* (Pedersen 2020) framework for customizable visualization to produce publication-ready figures. In addition, the package utilizes the basic data structure and features of *Seurat*, allowing users to employ well-known functions for data processing and visualization. The core of the analysis and visualization methods provided in *semla* is built upon an easily accessible S4 object intended for storing spatial and image data.

**Figure 1.**
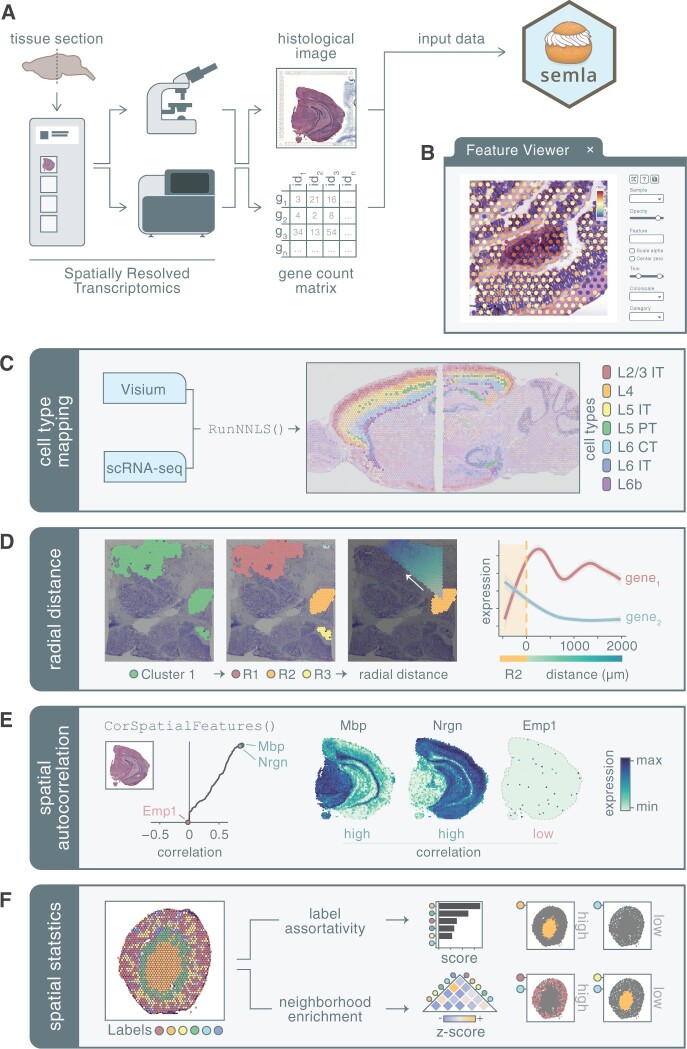
(A) The input for the *semla* R package consists of transcriptomics data and a matched histological image as generated by the Visium protocol and data processing pipeline. (B) A web application (“Feature Viewer”) can be opened through the R console and enables interactive exploration and annotation of the spatial expression data. Within *semla*, the user can apply various analyses to the data such as (C) cell type mapping using a matching single-cell RNA sequencing (scRNA-seq) dataset, (D) exploring gene expression along a radial distance from a defined region of interest, (E) detect spatially correlated genes across the entire sample, and (F) apply spatial statistics to describe spot label patterns.

As input, *semla* requires data generated with the Visium Gene Expression profiling platform, including expression matrices, histological images, and spot coordinate files produced with the 10x Genomics Space Ranger pipeline (Fig. 1A). Although *semla* is a Visium-centric toolkit in its current state, the framework is designed to allow for future support to load any spatial dataset represented by a feature × spot matrix and spot coordinates. *Semla* simplifies the task of combining and processing data from various tissue sections taken from different Visium capture areas. Users have the option to initiate web-based applications that facilitate interactive investigation and annotation of the data. This is especially handy when working with consecutive tissue sections that vary in sample positioning and orientation. An assortment of functions is available in *semla* to perform quality control, data processing, advanced spatial visualizations, cell type deconvolution, digital unrolling, and spatially aware analyses. Further descriptions of a selection of functions can be found in the Supplementary Information document and all documentation of the utilities available within *semla* is available at the package website (https://ludvigla.github.io/semla/), along with detailed tutorials which describe how these tools can be applied to real examples.

The package is available for installation through GitHub, https://github.com/ludvigla/semla.

## 3 Highlighted features

### 3.1 Interactive viewer

R is a popular programming language for SRT data analysis and offers a rich ecosystem of libraries to process and analyze data; however, interactive features are limited. For specific SRT analytical tasks, it is desirable to explore the data interactively and annotate spatial data in conjunction with the histological tissue image. A few alternative applications are available for this purpose (e.g. the Loupe Browser and spatialLIBD), though they are limited in their ability to seamlessly be integrated in the full analytical workflow. To address these needs, we have built an integrated web-based application within *semla* that facilitates interactive mapping of expression profiles and tissue annotations visualized onto the histological image of the tissue section (Fig. 1B, Supplementary Section S3.1). The interactive viewer is written in javascript using the popular UI library React, incorporated in *semla* using the *reactR* R package (Inc *et al.* 2021). The application is initiated within the R session and thereafter opens a web browser where it runs effortlessly when moving and zooming across the tissue image, facilitated by image tiling which is powered by the javascript-based web viewer OpenSeadragon. All numerical variables residing within the *Seurat* object, including gene expression values or dimensionality reduction vectors, are accessible for mapping on the tissue sections. Moreover, the viewer includes a lasso tool, with which users can select data points based on tissue morphology or feature expression and label these selections for downstream analysis.

### 3.2 Cell type mapping with NNLS

In Visium, the measured transcription profile in each spot typically represents mixed signals from multiple cells. Computational methods have been developed to deconvolve mixed expression profiles to predict the cell type composition (Andersson *et al.* 2020, Elosua-Bayes *et al.* 2021, Cable *et al.* 2022, Kleshchevnikov *et al.* 2022). Many of these methods require high performance computers and may take hours to run on moderately large datasets. *Semla* includes an approach for cell type deconvolution based on Non-Negative Least Squares (NNLS), which runs in a matter of seconds on Visium data with up to 100 000 spots (Fig. 1C, Supplementary Section S3.2). In short, the first step is to estimate cell type enrichment scores by comparing the ratios of averaged gene expression levels between a cell type of interest and the other cell types in the scRNA-seq data. These scores describe relative differences in expression levels between the cell types and are subsequently leveraged into the NNLS method to estimate the proportions of cell types in each Visium spot. The enrichment scores for each cell type are combined into a matrix **A**. Given the matrix **A** and a mixed expression profile **y** for a Visium spot, the NNLS method solves the following problem:

(1) $${\arg\;\min}_{x}{\left|\left|Ax-y\right|\right|}_{2}^{2},\;\mathrm{subject}\;\mathrm{to}\;x\geq 0,$$

where the solution for **x** represents the fractional abundances of cell types in the mixed expression profile. As a final step, the values for **x** are converted into cell type proportions. To assess the performance of our deconvolution method, we benchmarked NNLS against four other methods: RCTD, *stereoscope*, *cell2location*, and Seurat label transfer. Based on our benchmark results, we could conclude that the cell type proportions estimated with NNLS, RCTD, *stereoscope* and *cell2location* correlated well with the expected proportions. RCTD and *cell2location* demonstrated the highest accuracy, whereas the NNLS method outperformed all other methods in terms of computational speed. The full performance comparison is available in Supplementary Section S3.3 and on the *semla* package website.

### 3.3 Spatially aware analyses

To take full advantage of the spatial component in SRT data, computational tools can incorporate a distance or connectivity parameter to model the spatial relationships between spots. We have implemented a set of methods in *semla* to describe spatial relationships and identify new spatial patterns. A set of helper functions are also available for the user to explore and address their own spatially related hypotheses more easily. As an example, we can, in a few steps, split collections of spots into spatially disconnected regions, identify their immediate border zone, or study gene expression changes in an outwards trajectory from the border at different angles (Fig. 1D).

A fast implementation for identifying genes with spatial variability is available in *semla*, which builds on the method described by Bergenstrahle *et al.* (2020), is available in *semla* (Fig. 1E). In short, the method ranks genes based on the Pearson correlation between each gene’s expression vector across spots and the averaged expression vector across the spots’ immediate spatial neighbors. A high correlation score for a gene is associated with a tendency for regions close together in space to have similar values for that gene. In contrast, genes with low scores have more random expression levels in the tissue section, indicating little or no spatial structure. These scores may be used for feature selection to focus downstream analytical tasks on genes that exhibit spatial structure in the data.

In more unorganized tissues, it may be desirable to statistically describe the spatial relationships of spots assigned with labels. *Semla* includes two approaches for computing spatial statistics of labeled spots: neighborhood enrichment and label assortativity (Fig. 1F, Supplementary Section S3.4). The neighborhood enrichment test describes how often spots of two different categories lie next to each other and vice versa, which is used to identify and quantify co-occurring structures, while the label assortativity test measures the connectivity between spots sharing the same label and can therefore be used to describe whether spots of a given category displays an aggregated or dispersed spatial pattern in the sample.

### 3.4 Conclusion

Here, we have presented *semla*, a versatile R package that delivers tools for Visium SRT data processing, analysis, and visualization. Created to accommodate biologists and bioinformaticians with variable programming skills, *semla* enables interactive examination of expression patterns within a histological context and provides sophisticated techniques for exploratory analysis.

## Supplementary Material

btad626_Supplementary_Data
