# Supplementary material for: Semla: a versatile toolkit for spatially resolved transcriptomics analysis and visualization
Source: Bioinformatics. 2023 Oct 16;39(10):btad626. doi: 10.1093/bioinformatics/btad626 (PMC10597621; doi:10.1093/bioinformatics/btad626)
Supplement: btad626_Supplementary_Data [file btad626_supplementary_data.pdf]

Supplementary Information

Semla: A versatile toolkit for spatially resolved transcriptomics analysis and visualization

Ludvig Larsson, Lovisa Franzén, Patrik L Ståhl, Joakim Lundeberg

2023

The supplementary information to the main article includes:

1. Package dependencies....2
2. Operating System testing....3
3. Selected function descriptions....3
3.1. Interactive applications....3
3.2. NNLS cell type mapping methodology description....6
3.3. Benchmarking the NNLS methodology....7
3.4. Label Assortativity and Neighborhood Enrichment....16
3.5. Digital unrolling....17
4. Comments on *semla* and other tools for SRT analysis and exploration....21
5. Limitations....24
6. References....26

1. Package dependencies

These are the following package dependencies, imports, and suggestions for *semla* (version 1.1).

**Depends**	R (>= 4.1.0),<br>Seurat (>= 4.0.0),<br>SeuratObject (>= 4.0.0),<br>dplyr (>= 1.0.0),<br>ggplot2 (>= 3.3.0)
**Imports**	cli,<br>forcats,<br>jsonlite,<br>rlang,<br>tibble,<br>tidyr,<br>methods,<br>glue,<br>magick (>= 2.7.0),<br>Matrix (>= 1.5-0),<br>patchwork (>= 1.1.0),<br>scales (>= 1.2.0),<br>zeallot,<br>dbscan (>= 1.1.0),<br>RColorBrewer,<br>shiny,<br>shinyjs,<br>reactR
**Suggests**	farver,<br>tidygraph,<br>igraph,<br>leaflet,<br>viridis,<br>testthat (>= 3.0.0),<br>data.table,<br>BiocManager (>= 1.30.18),<br>MatrixExtra,<br>htmlwidgets,<br>htmltools,<br>RcppML,<br>beakr,<br>colourpicker,<br>fs,<br>shinyBS,<br>ggnewscale,<br>ggsci,<br>scico,<br>ggfittext<br>hdf5r

2. Operating System testing

The *semla* R package was developed using MacOS and has been further tested on multiple operating systems (OS) to ensure it can be installed correctly and functions without error. OS unavailable to the development team for local testing were tested using the R package "rhub" (Csárdi et al. 2023) for remote checks using the R-hub builder.

The following OS have been tested:

- macOS Big Sur 10.16 install (devel), R version 4.2.1 (local test)
- macOS Catalina 10.15.7 install (devel), R version 4.1.2 (local test)
- macOS Monterey 12.3.1 install (devel), R version 4.2.1 (local test)
- macOS Ventura 13.4 install (devel), R version 4.1.2 (local test)
- Windows 10 Education, 64-bit (local test)
- Fedora Linux, R-devel, clang, gfortran (rhub test)
- Ubuntu Linux 20.04.1 LTS, R-release, GCC (rhub test)
- Windows Server 2022, R-devel, 64 bit (rhub test)

3. Selected function descriptions

3.1. Interactive applications

Usage description

We have built a web-based application called the Feature Viewer, that allows interactive exploration and labeling of the SRT data set. The application is written in javascript and uses the React UI library. Some of the functionality is similar to what you can do with the Loupe Browser (10x Genomics) or the ST viewer (Fernandez Navarro et al. 2019). In comparison to interactive user interfaces provided in other R packages for Visium data analysis (SpatialLIBD (Pardo et al. 2022), Seurat (Hao et al. 2021), SPATA2 (Kueckelhaus et al. 2023), Giotto (Dries et al. 2021)), the Feature Viewer in *semla* allows users to interactively explore the histology image in higher resolution at different levels of magnification, select among thousands of features to plot, easily switch between the available samples, and use a selection tool to annotate spots and save them as labels for downstream analysis.

To use the Feature Viewer, the user first needs to import their SRT data with *semla* and further load the coupled histology image, using the `LoadImage()` function. Once the data of interest is available in R, the Feature Viewer can be initiated with the *semla* function `FeatureViewer()`,

```
Unset
se <- FeatureViewer(se)
```

where “se” represents a Seurat object compatible with *semla*. Running this command within an R environment will open a new web browser window containing the interactive user interface with your data. If using a very large image, it is possible to speed up the process of engaging the Feature Viewer by tiling the image beforehand using the `ExportDataForViewer()` function (this process can take some time, read under *Performance* for additional information). Supplementary Figure 1 illustrates the user interface of the Feature Viewer application.

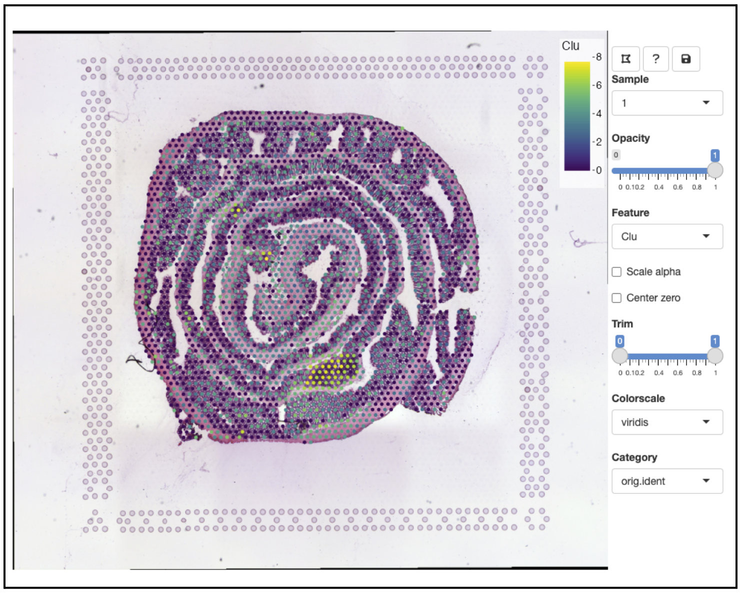

Supplementary Figure 1. *Feature Viewer used to visualize the mouse colon demo data set available within semla.*

To save and exit the Feature Viewer, the user needs to press the “save & quit” icon and may thereafter close the browser window and return to the R session. Any annotations made with the lasso tool will be saved as additional metadata columns in the Seurat object when the Feature Viewer is closed. The R session will be occupied as long as the Viewer is active.

In addition to the Feature Viewer application, *semla* provides another interactive user interface (UI) for aligning samples (Supplementary Figure 2). With the alignment application, the user can perform

transformations such as rotations, mirroring, and moving to the images of multiple tissue sections. The tool is available through the `RunAlignment()` function,

```
Unset
se <- RunAlignment(se)
```

More details on how to use the alignment tool is available on the *semla* tutorial website https://ludvigla.github.io/semla/articles/image_alignment.html.

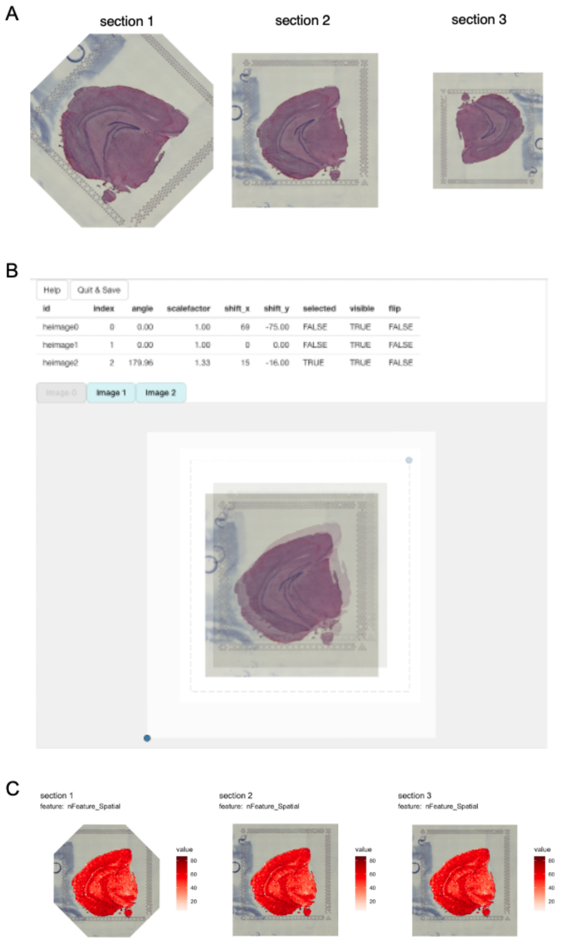

Supplementary Figure 2. *Example case of aligning three tissue sections to achieve matching tissue orientation and size. A) Sections 1-3 before alignment. 2) The interactive alignment application is used to adjust the individual images before saving the transformations. 3) The newly aligned images can be used for generating spatial feature plots.*

Performance

The Feature Viewer employs image tiling, through the `TileImage()` function, to enhance interactivity with H&E images. The computation time for the tiling step depends on the number of samples, the desired number of zoom levels and the size of the input images. For a single tissue section data set with an H&E image approximately 2,000x2,000 pixels large, the tiling step with `TileImage()` completes in a few seconds. For reference, in a test dataset, the tiling process took approximately 3 seconds to complete on a MacBook Pro laptop (2017 model, 3.1 GHz Quad-Core Intel Core i7, 16GB RAM). `TileImage()` automatically determines the suitable number of zoom levels based on the input image's size, for instance, a 2,000x2,000 H&E image results in the creation of three layers containing tiles of sizes 2x2, 4x4, and 8x8. Once the tiling step is completed, the tiles are stored locally for future use and the viewer can be launched almost instantaneously in the default browser.

For executing the image tiling separately, a utility function named `ExportDataForViewer()` can be employed where an option to accelerate the tiling process by utilizing multiple threads is available. When dealing with larger H&E images and/or multiple tissue sections, a greater number of tiles must be exported, thus extending the overall processing time. The image tiling performed through this approach is a one-time operation, and will therefore allow the Feature Viewer to be engaged quickly for as long as the exported tiled images are available. Upon supplying the tiles, the `FeatureViewer()` function can be invoked to promptly open the viewer, which launches almost instantaneously within a web browser.

3.2. NNLS cell type mapping methodology description

Semla includes an approach, based on Non-Negative Least Squares (NNLS), for inferring cell type proportions in each spot. The method requires paired Visium and annotated scRNA-seq data generated from the same source. In short, the method first estimates cell type enrichment scores from the annotated cells in the scRNA-seq data by comparing their normalized and averaged gene expression profiles. This scoring scheme assigns higher weights to genes that are cell type specific, thereby providing a profile that describes relative differences between the cell types. The enrichment profiles are subsequently leveraged into the NNLS method to predict the composition of cells in the Visium data. Given the Visium gene expression matrix **A** and the cell type enrichment profiles **y**, the NNLS method attempts to solve the following problem:

$$arg\ min_x ||Ax - y||_2^2, \text{ subject to } x \geq 0$$

where the solution for x represents the cell type estimates in the Visium data.

Within *semla*, the NNLS cell type mapping is executed by running the `RunNNLS()` function, providing the spatial object of interest along with a normalized scRNA-seq Seurat object and specifying the metadata column containing the cell group annotations:

```
Unset
se_spatial <- RunNNLS(object = se_spatial,
                      singlecell_object = se_allen,
                      groups = "subclass")
```

With a reasonably sized data set, the analysis should finish in a matter of seconds. The resulting cell type proportion estimates are stored in a new assay, “celltypeprops”, residing within the spatial object, that is easily accessible for downstream exploration and visualization.

3.3. Benchmarking the NNLS methodology

To assess the utility of the NNLS cell type mapping approach, we have performed a few comparisons of NNLS with other established cell type mapping methods in order to benchmark its performance. The articles outlining the comparisons and containing the code for reproducing the plots are accessible on the *semla* website.

Publicly available Visium data

Initially, we tested the performance of NNLS, *stereoscope* (Andersson et al. 2020), and *cell2location* (Kleshchevnikov et al. 2022) using publicly available Visium data sets and tissue type matched single cell RNA-sequencing (scRNA-seq) data sets, all publicly available (Supplementary Table 1). *Stereoscope* and *cell2location* were run in python on high-performance computing servers using default parameter settings, while NNLS was run locally on a laptop. Examining the Pearson correlation between the inferred cell type compositions between the different methods, we could observe high concordance for both tested tissue types (Supplementary Figure 3). Unfortunately, the ground truth of the actual cell type compositions in each spot of the Visium data is unknown, making it impossible to evaluate the actual accuracy of the NNLS method with these data sets. However, both *stereoscope* and *cell2location* are two well established methods for estimating cell type abundances, and thus we deem the NNLS approach to produce comparable results.

Tissue type	scRNA-seq data set	Visium data set
Mouse brain	Allen Brain, mouse atlas reference single cell RNA-seq data set (Tasic et al., 2016) https://doi.org/10.1038/nn.4216	Mouse brain sagittal data (anterior + posterior) made available by 10x Genomics, processed using the Space Ranger pipeline v1.0.0. https://www.10xgenomics.com/cn/resources/datasets/mouse-brain-serial-section-1-sagittal-anterior-1-standard-1-0-0
Mouse kidney	Tabula Muris Senis droplet data from kidney, made available by The Tabula Muris Consortium. (Tabula Muris Consortium, 2020) https://doi.org/10.1038/s41586-020-2496-1	Mouse kidney coronal section data made available by 10x Genomics, processed using the Space Ranger pipeline v1.1.0. https://www.10xgenomics.com/cn/resources/datasets/mouse-kidney-section-coronal-1-standard-1-1-0

Supplementary Table 1. *Data sets used for cell type mapping.*

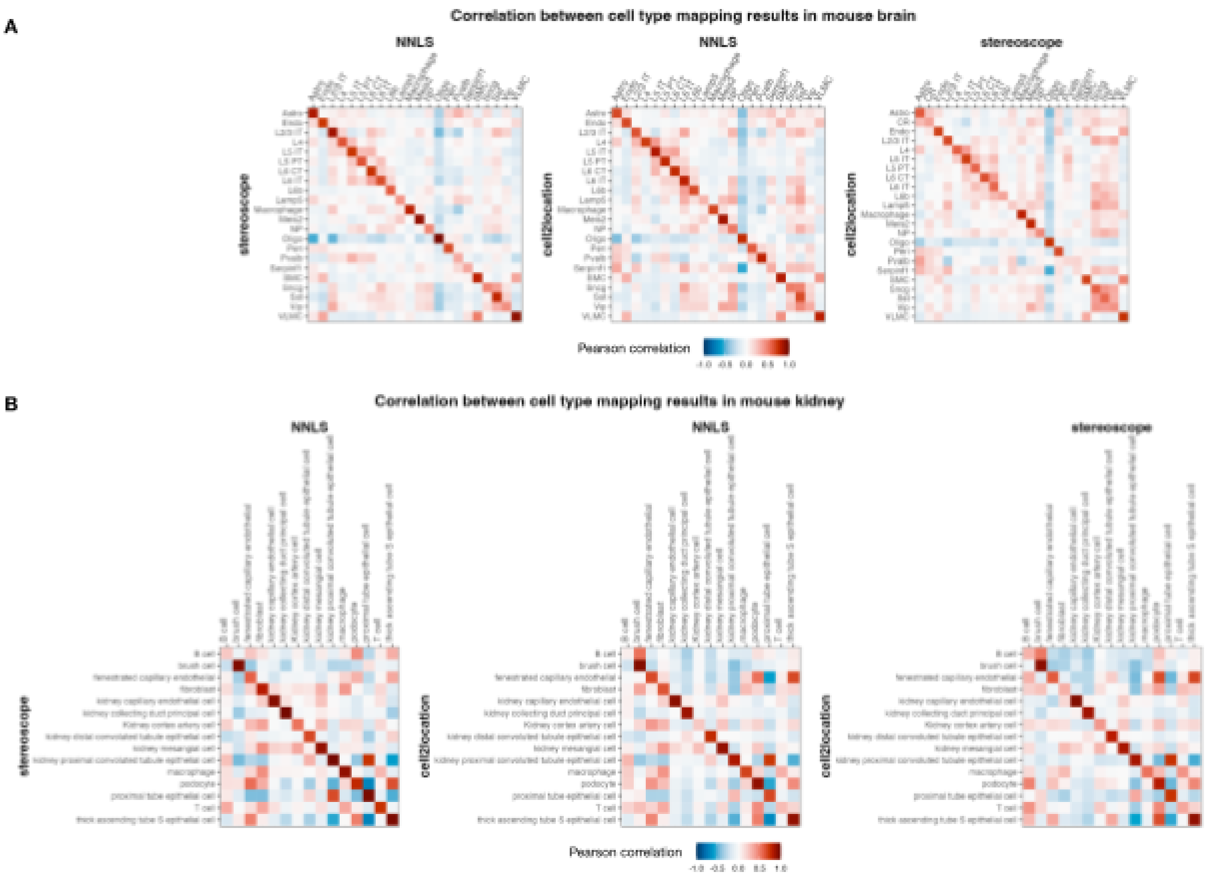

Supplementary Figure 3. *Comparison of output from cell type mapping algorithms NNLS, stereoscope, and cell2location, on mouse brain (A) and mouse kidney (B) data sets, which demonstrates overall high Pearson correlation values for most cell types.*

Synthetic data

To evaluate the performance of the NNLS approach using a data set where the ground truth of cell type proportions per spot is available, we prepared a synthetic Visium data set. In order to make the synthetic data set representative of a typical Visium data set, we opted to generate synthetic spots with an average of 10 cells and with a median of 20,000 UMIs per cell. A count matrix from a single-cell RNA-seq (Smart-seq 2) data set obtained from the Allen Brain atlas (Tasic et al., 2016) with 14,249 cells was used to create the synthetic spots. Only the top 5,000 most variable genes were kept in the UMI count matrix to speed up computation run time. Based on the distribution of UMI counts per cell in the scRNA-seq data set, we downsampled the count matrix to 1% using the downsampleMatrix function from the scuttle R package. Next, we generated cell counts by drawing 10,000 random numbers from a poisson distribution with the mean set to 10 (number of cells per spot). Zero values were replaced with a value of one to make sure that each synthetic spot would include at least one cell type. Each synthetic spot was created by sampling and aggregating N random cell expression vectors, where N represents the number of cells per spot. Sampling probabilities were biased to reflect the composition of cell types in the scRNA-seq data set, thereby ensuring that the abundances of cell types in the scRNA-seq data set was reflected in the synthetic Visium data. The ground truth corresponds to the proportion of cell type labels for each synthetic spot. For run time performance assessment (NNLS and Seurat label transfer), we used the same approach to create a large synthetic data set with 100,000 spots. For all benchmark analyses, the scRNA-seq data set was downsampled to include a maximum of 250 cells per cell type and filtered to exclude cell types with fewer than 10 cells.

NNLS

The NNLS technique was executed using a cap of 250 cells per cell type as the upper limit and a minimum of 10 cells per cell type. In order to evaluate runtime performance, the NNLS approach was employed in 10 cycles, deconvolving expression data for 10,000 to 100,000 spots in the large synthetic data set (Supplementary Figure 4). All computations were run on a Macbook Pro (2017, 3.1 GHz Quad-Core Intel Core i7, 16GB).

Seurat label transfer

The Seurat label transfer method was used to calculate cell type prediction scores from the synthetic Visium data following the Seurat tutorial on integration with ‘single-cell’ data. The standard logNormalize method was used to normalize the UMI count matrix. For run time performance assessment, the Seurat label transfer method was run in 10 iterations, deconvolving the mixed expression

data for 10,000 to 100,000 spots in the large synthetic data set. All computations were run on a Macbook Pro (2017, 3.1 GHz Quad-Core Intel Core i7, 16GB).

RCTD

The RCTD method was run using standard parameter settings following their RCTD tutorial on Visium data, with the doublet mode set to 'full'. Seven cores were used for the computation. For the RCTD deconvolution, a Macbook Pro (2017, 3.1 GHz Quad-Core Intel Core i7, 16GB) was used.

Stereoscope

For deconvolution with *stereoscope*, we used the scVI (https://scvi-tools.org/) implementation, following the tutorial 'STereoscope applied to left ventricule data'. First, a model was trained on the scRNA-seq data with 1,000 epochs. Next, the model was used to deconvolve the synthetic Visium expression profiles in 2,000 epochs. For the *stereoscope* deconvolution, we used a NVIDIA A100-SMX4-80GB Tensor core GPU.

Cell2location

For deconvolution with *cell2location*, we followed their tutorial 'Mapping human lymph node cell types to 10X Visium with *cell2location*'. First, a model was trained on the scRNA-seq data with 1,000 epochs. Next, the model was used to deconvolve the synthetic Visium expression profiles in 30,000 epochs. For the *cell2location* deconvolution, we used a NVIDIA A100-SMX4-80GB Tensor core GPU.

NNLS computation time

The NNLS method can be used to deconvolve Visium data in a matter of seconds, even for larger data sets. Supplementary Figure 4 shows that the average computation time for 100,000 spots is 4 seconds.

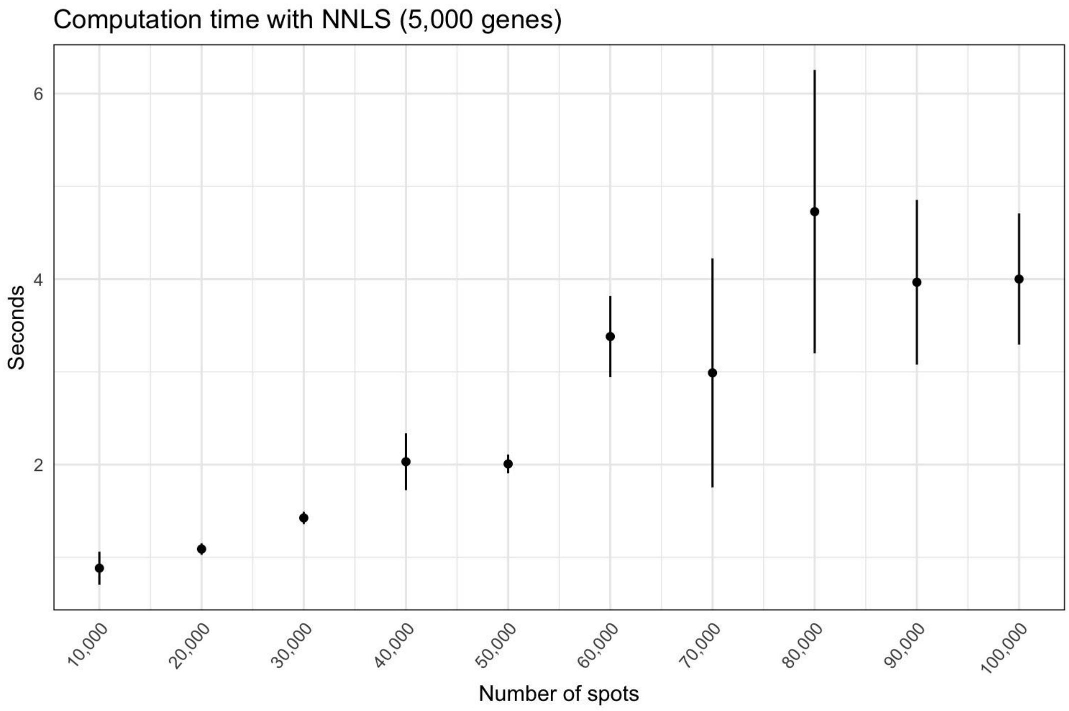

Supplementary Figure 4. *Computation time for NNLS on synthetic Visium data. The x-axis shows the number of spots included in the synthetic Visium data and the y-axis indicates the computation time in seconds. Each data point represents the average computation time across 10 iterations. The whiskers indicate the standard deviation.*

NNLS vs Seurat label transfer* computation *time

The second fastest method included in this benchmark is the Seurat label transfer method used for cell type prediction. Although not a pure deconvolution method, this technique facilitates the probabilistic transfer of labels from a reference (single-cell) to a query (Visium) dataset. As depicted in Supplementary Figure 5, it's evident that the NNLS method consistently outperforms the Seurat label transfer method in terms of speed.

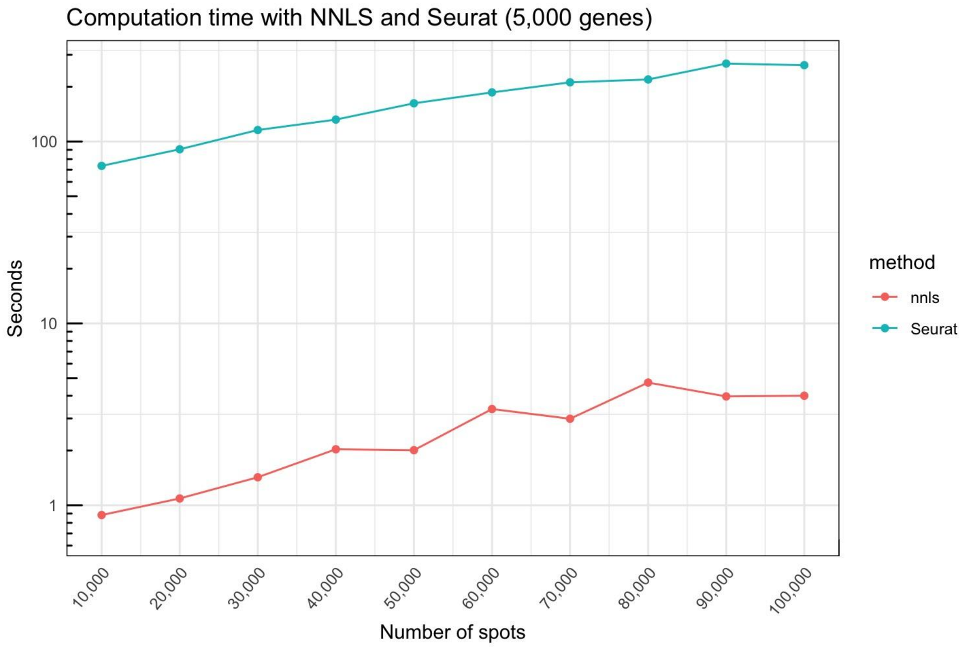

Supplementary Figure 5. *Computation time for NNLS and the Seurat label transfer method on synthetic Visium data. The x-axis shows the number of spots included in the synthetic Visium data and the y-axis indicates the computation time in seconds.*

Computation time for 10,000 spots

Although NNLS, Seurat label transfer, and RCTD could be executed on a laptop using 10,000 spots, both *stereoscope* and *cell2location* necessitated a high-performance GPU for operation. Due to the substantial computational demands, RCTD, *stereoscope*, and *cell2location* were assessed only with 10,000 spots, a process taking several hours to complete. Supplementary Figure 6 illustrates the superior computational speed of the NNLS method compared to all other techniques included in the comparison.

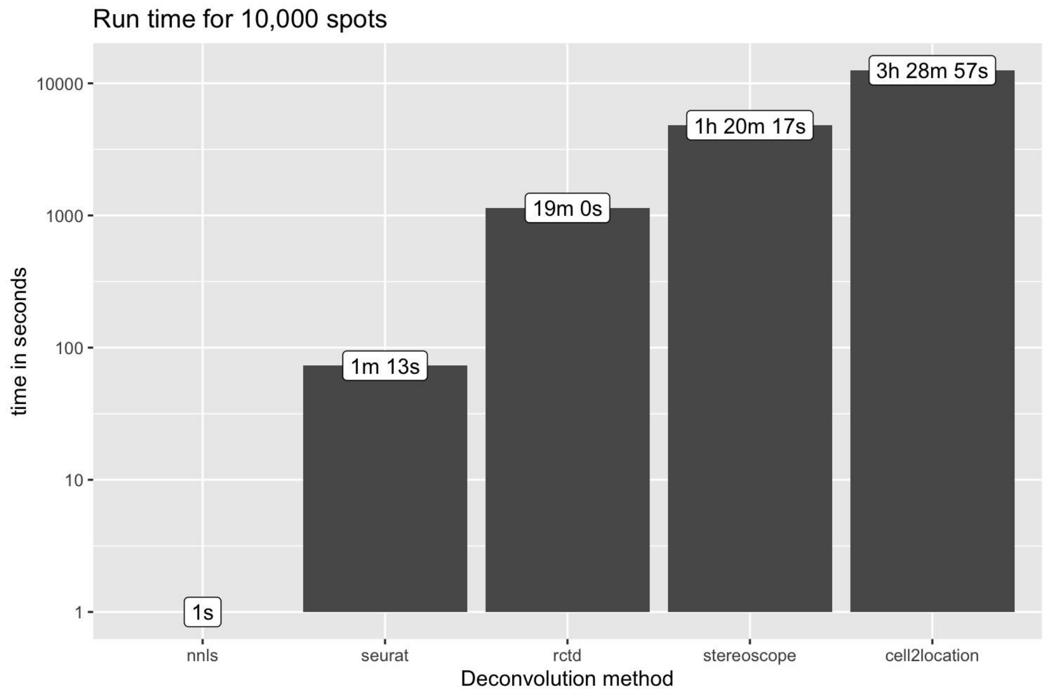

Supplementary Figure 6. *Computation time for NNLS, Seurat label transfer, RCTD, stereoscope and cell2location. The y-axis shows the computation time in seconds (*log_{10} *scale).*

Performance assessment

In the assessment of method precision, we employed two performance metrics: Pearson correlation and root mean square error (RMSE), visualized in Supplementary Figures 7-9. The Pearson correlation scores highlight that RCTD and *cell2location* present the most robust correlation regarding the inferred and estimated cell type proportions. Conversely, NNLS and *stereoscope* exhibit slightly diminished correlation concerning specific cell types. The Seurat label transfer method generally showcases reduced correlation values across all cell types. The RMSE values follow a comparable pattern, with NNLS, RCTD, *stereoscope*, and *cell2location* demonstrating similar performance.

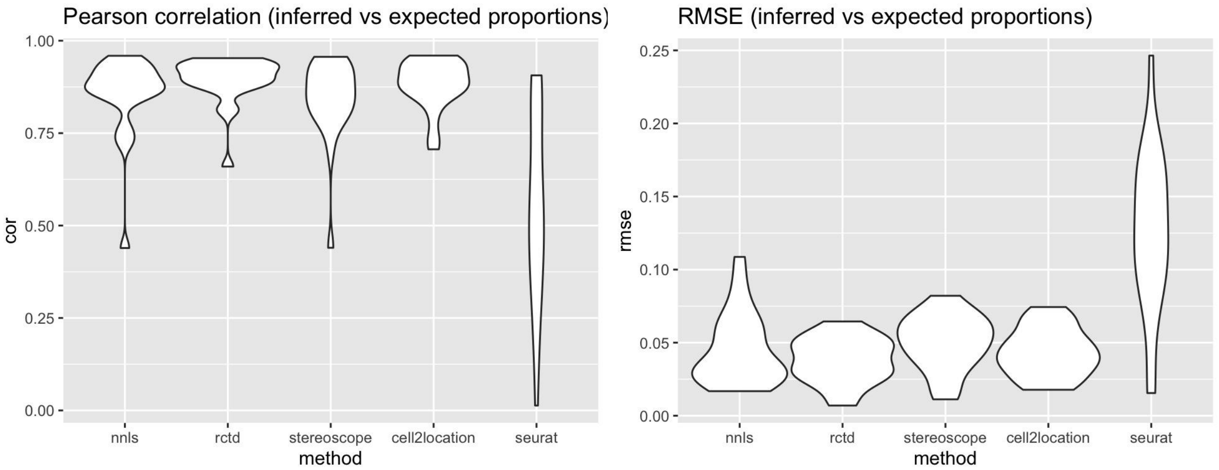

Supplementary Figure 7. *Performance assessment metric for NNLS, Seurat label transfer, RCTD, stereoscope and cell2location on 10,000 synthetic spots. Left: Pearson correlation between inferred and expected proportions for all cell types. Right: RMSE between inferred and expected proportions for all cell types. RMSE measures the average difference between the inferred proportions and the actual proportions.*

Supplementary Figure 8 depicts the contrast between inferred and expected cell type proportions for 22 cell types across the five deconvolution methods tested. RCTD, *stereoscope*, and *cell2location* exhibit a generally strong agreement between the inferred and expected proportions, although there's a consistent tendency to overestimate proportions for certain cell types like astrocytes and macrophages. NNLS demonstrates skewed proportion estimations for various cell types in comparison to RCTD, *stereoscope*, and *cell2location*. This includes specific excitatory neurons within the mouse visual cortex (L2/3 IT, L6 CT, and L6 IT), Pvalb+ neurons, and Lamp5+ neurons. Nonetheless, NNLS yields slightly improved proportion estimates for other cell types such as astrocytes, endothelial cells, macrophages, and oligodendrocytes. Overall, the proportions estimated with NNLS exhibit a strong correlation with the expected proportions, suggesting that the spatial distribution of cell types remains dependable, even if the abundance of certain cell types might be less reliable. In line with previous observations, the Seurat label transfer method exhibits the worst concordance across all cell types.

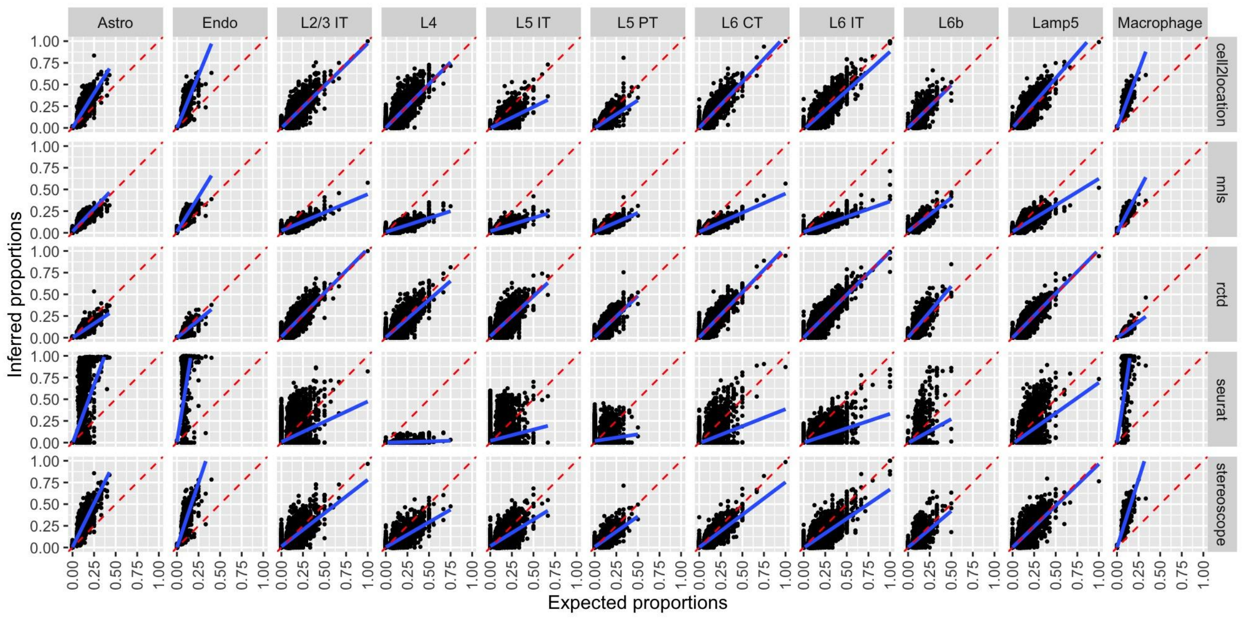

Supplementary Figure 8. *Inferred vs expected proportions for the* _first_ *11 cell types. Each data point corresponds to a synthetic spot. The blue lines highlight a fitted trend line and the dashed lines indicate the expected 1 to 1 relationship.*

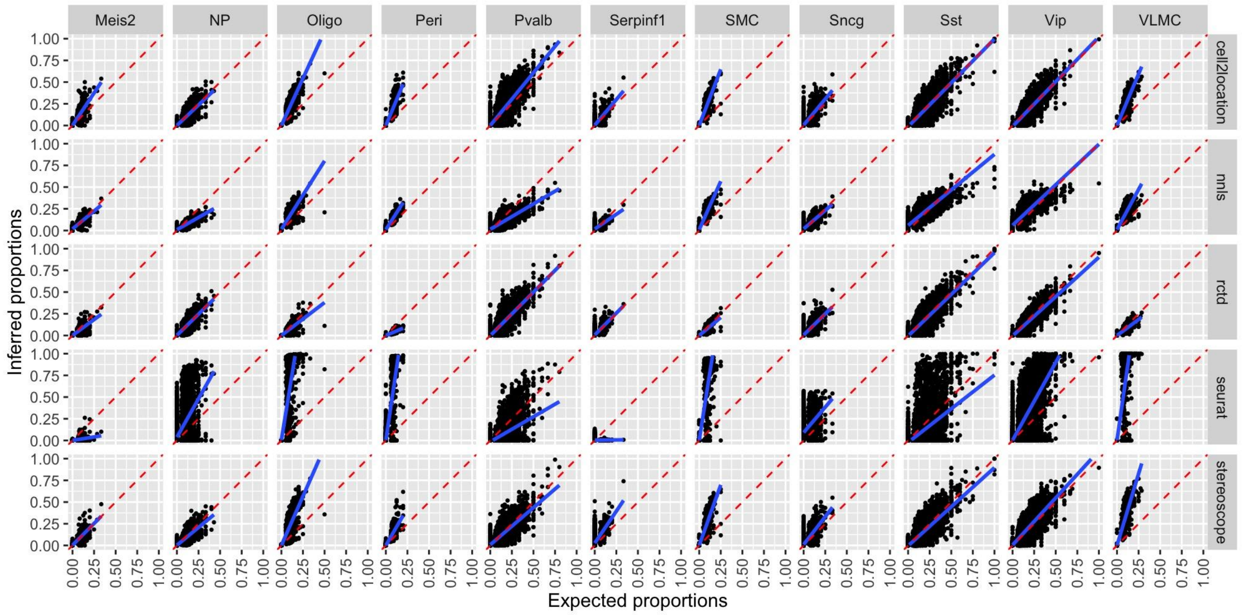

Supplementary Figure 9. *Inferred vs expected proportions for the* _last_ *11 cell types where each data point corresponds to a synthetic spot. (Blue lines: fitted trend line, dashed lines: expected 1 to 1 relationship)*

Conclusion

Our benchmark analyses on synthetic Visium data indicate that the NNLS approach generates results that are comparable with RCTD, *cell2location*, and *stereoscope*. Notably, in contrast to these approaches, NNLS operates within a matter of seconds for datasets of up to 100,000 spots, without the need for hardware acceleration. RCTD and *cell2location* generated more accurate proportion estimates compared to NNLS and we therefore encourage users to test these more robust and well-established cell type deconvolution algorithms. Nonetheless, we do recognize that NNLS presents a viable alternative for fast cell type decomposition, which proves particularly beneficial during various stages of exploratory data analysis.

For instance, cell type deconvolution of SRT data often necessitates careful curation of the single-cell reference data set to make sure that the cell types included for deconvolution are present in the tissue section. This can be particularly challenging when working with large atlases which encompass whole organs or even multiple organs. Additionally, creating cell atlases from scRNA-seq data entails iterative refinement of quality filters and tuning of hyperparameters. Cell type deconvolution offers valuable insights for informing these choices by spatially mapping the cell types, yet this iterative process can be hindered by computation time and hardware constraints. With a fast deconvolution approach such as NNLS, this curation step becomes more time efficient and accessible to users who are not familiar working with high performance machines. Once the foundation of a single-cell reference is laid, users can subsequently employ a more robust deconvolution technique to attain enhanced accuracy in their proportion estimates.

3.4. Label Assortativity and Neighborhood Enrichment

The term *assortativity* is used within network science to describe the connectivity between nodes of similar properties. Traditionally, this is measured in terms of the node's degree and computing a correlation coefficient between nodes of similar degrees. A few methods based on this are Newman's Assortativity (Newman, 2002) and Ripley's Function (Ripley, 1976). Inspired by this, we have developed a straightforward approach to estimate the connectivity of spots belonging to the same cluster, i.e. sharing the same label, by measuring the network's average degree, $\langle k \rangle$, for each label and comparing this to a completely randomly dispersed pattern. The randomly distributed pattern can be viewed as the baseline, since we rarely obtain a pattern more dispersed than that, while a group of spots that is fully connected with each other is the highest order of organization we can achieve. In this method, each label's $\langle k \rangle$ is therefore min-max scaled between the $\langle k \rangle$ of a randomized network (min) and the $\langle k \rangle$ of the fully connected network (max). The output from this analysis, executed using the

`RunLabelAssortativityTest(se)` function, is a table containing a scaled average degree for each unique label provided for the analysis, along with stored values of the intermediates used for the calculations of the final score. The scaled average degree ranges between ~0-1, with values around 0 being equal to a randomly dispersed spatial pattern and values closer to 1 indicating an aggregated and highly connected organization of the spots of that label.

The purpose of a neighborhood enrichment analysis on the other hand is to test whether spots belonging to two different categories are localized next to each other spatially. To estimate the enrichment of their co-localization we compare it against random permutations of the labels, forming the null hypothesis stating that the spots of the two labels are distributed randomly and share no connections other than those observed by chance. A z-score for each label pair is calculated as:

$$Z_{AB} = \frac{x_{AB} - \mu_{AB}}{\sigma_{AB}}$$

where x_{AB} is the number of edges observed between spots of labels A and B, μ_{AB} is the permutation mean of the edges between A and B, and σ_{AB} is the permutation standard deviation of the edges between A and B. Thus, a z-score of around 0 can be interpreted as a spatial label co-localization equal to that seen by chance, given the number of spots within those categories, while a positive z-score indicates an over-representation of the label pair proximity and a negative z-score can be viewed as a depletion, or repellant effect, of the label pair spatially. The neighborhood enrichment analysis is run by calling the function `RunNeighborhoodEnrichmentTest()` and stores the results into an output table containing the z-scores between each label pair.

The Label Assortativity and Neighborhood Enrichment tests included in *semla* are implementations and further developments of the *heterotypic* and *homotypic scores* described in Bäckdahl et al (2021).

3.5. Digital unrolling

The "digital unrolling" approach was first presented by M. Parigi and colleagues (Parigi et al. 2022), developed to digitally unfold mouse colon samples rolled up into "swiss-rolls", enabling the analysis of gene expression variation along the proximal-distal axis of the organ. *Semla* includes a new strategy for "digital unrolling" that is conducted in two steps. In the first step, a spatial undirected network is created from the Visium spot coordinates, representing each spot as a node in the network where adjacent nodes are connected by an edge. The spatial network can then be visualized and modified using the `CutSpatialNetwork()` function from an R session, which opens an interactive tool in the web browser. The interactive tool overlays the spatial network on top of the histological H&E image of the

tissue section. Guided by the tissue histology, users can then cut edges between adjacent layers of the "swiss-roll" (Supplementary Figure 10). Once the edge cutting step is complete, the modifications are saved and returned to the R session for downstream processing. The selected edges are then filtered from the spatial network to produce a reduced network where the adjacent layers are disconnected (Supplementary Figure 11).

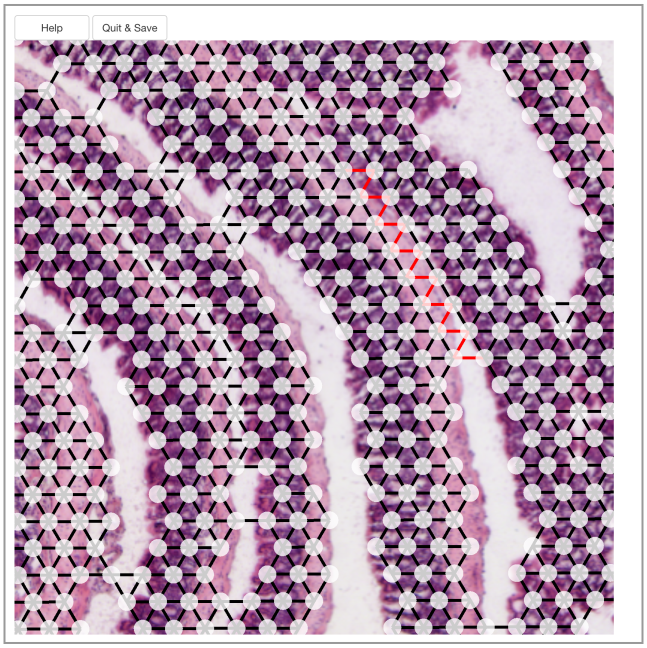

Supplementary Figure 10. *Example illustrating how edges can be cut in a spatial network using the* `CutSpatialNetwork()` *tool*.

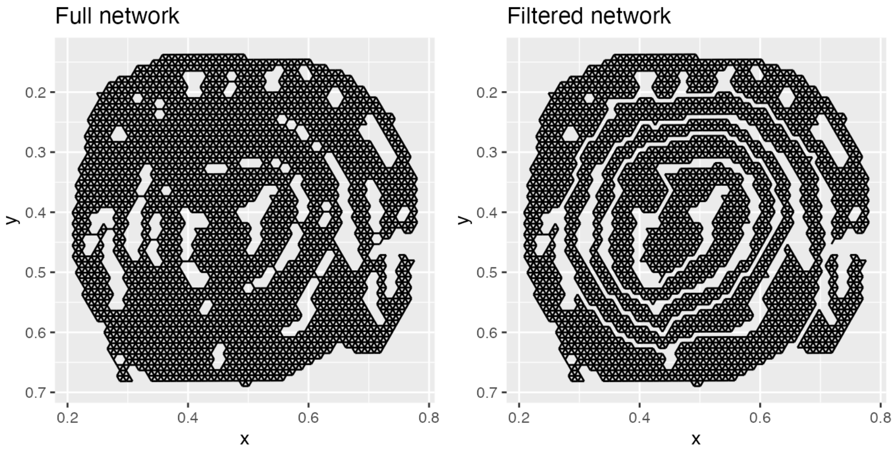

Supplementary Figure 11. *Example of a spatial network before and after edge filtering*.

The filtered spatial network can thereafter be used for the second step which aims to position spots along the proximal-distal axis of the “swiss-roll”. This “unfolding” step is done using the `AdjustTissueCoordinates()` function which takes the modified spatial network as input. Note that the unfolding step assumes that the spatial network is fully connected. If multiple disconnected network components are detected, the largest network component will be selected. The “unfolding” algorithm starts by identifying the pair of nodes (spots) separated by the largest distance in the network. These spots should correspond to the most proximal and distal nodes which serve as the start and end points of the x-axis in the unfolded coordinate system. Next, the algorithm identifies the shortest path between these end points, returning node IDs along this path. Each node along this path is assigned with an x coordinate determined by its order from start to end point. Subsequently, pairwise distances are calculated between all remaining nodes and the nodes on the shortest path. Each node is then assigned the same x coordinate as the closest shortest path node (Supplementary Figure 12) and the y coordinate corresponds to the geodesic distance to the closest shortest path node.

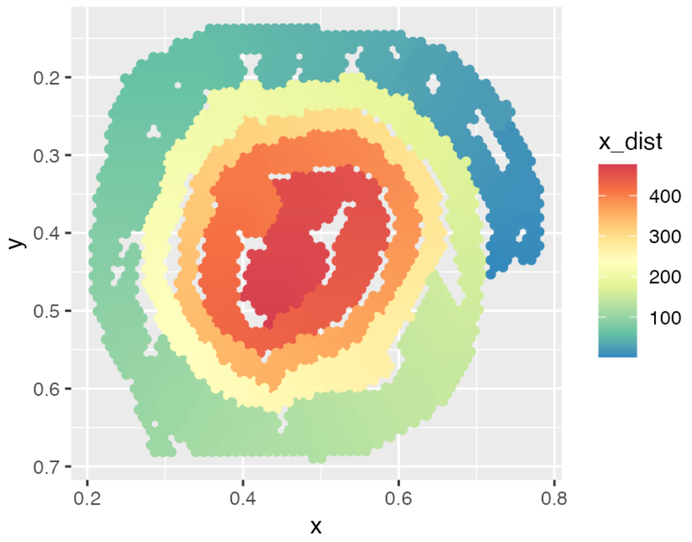

Supplementary Figure 12. *Spots colored by distance along the proximal-distal axis in the “unfolded” coordinate system.*

Subsequent downstream analyses and visualizations can leverage the new "unrolled" spot coordinate system to identify relevant trends along the proximal-distal axis. For instance, regionalization of cell type composition or marker gene expression. This concept is demonstrated in Supplementary Figure 13, where the expression patterns of four chosen marker genes along the proximal-distal axis are illustrated.

Furthermore, there exists the potential to model gene expression as a function of distance, facilitating data-driven extraction of spatial regionalization patterns along the proximal-distal axis.

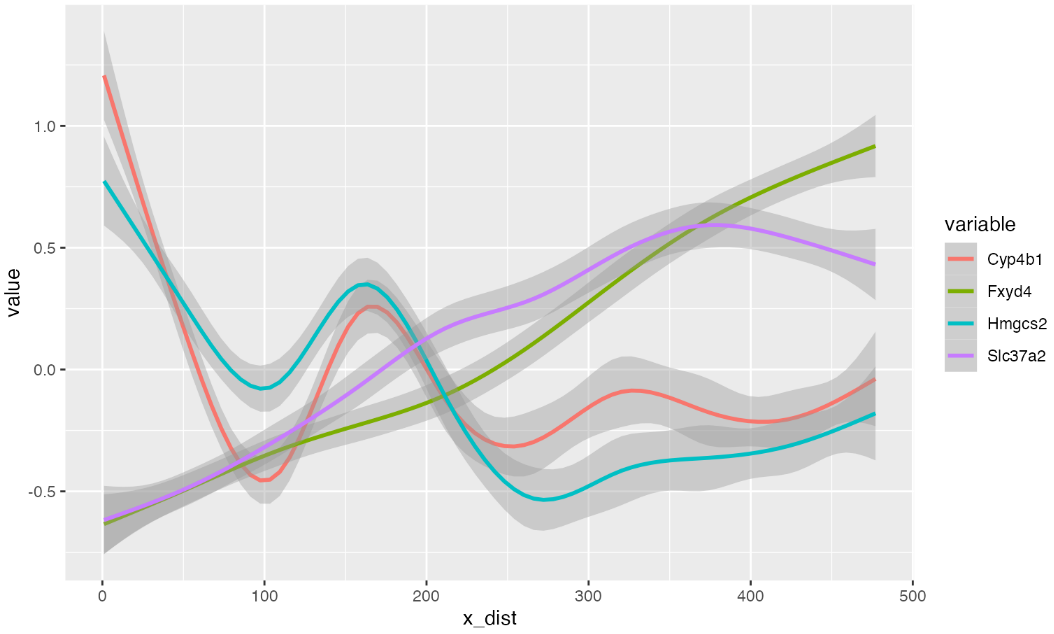

Supplementary Figure 13. *Gene expression trends for four selected marker genes along the proximal-distal axis of a mouse colon. The x-axis shows the distance along the proximal-distal axis. The y-axis shows normalized gene expression values.*

On a final note, the cutting tool for digital unrolling provided in *semla* could in theory be used to unfold other tissue types. For instance, in the small intestine the tool could be used to separate circular folds (*plicae circulae*) or individual villi. This extension could yield supplementary insights that could be harnessed to analyze and model spatial patterns within these specific structures. The "unrolling" algorithm, which takes a connected spatial network as input, makes certain assumptions about the shape of the tissue and is therefore only useful for special applications. However, it proves highly advantageous when tasked with swiftly computing distances between endpoints within a fully connected spatial network. As exemplified in Supplementary Figure 14, this approach is demonstrated in a scenario where spots were manually selected using the Feature Viewer tool, followed by distance calculations using the AdjustTissueCoordinates algorithm.

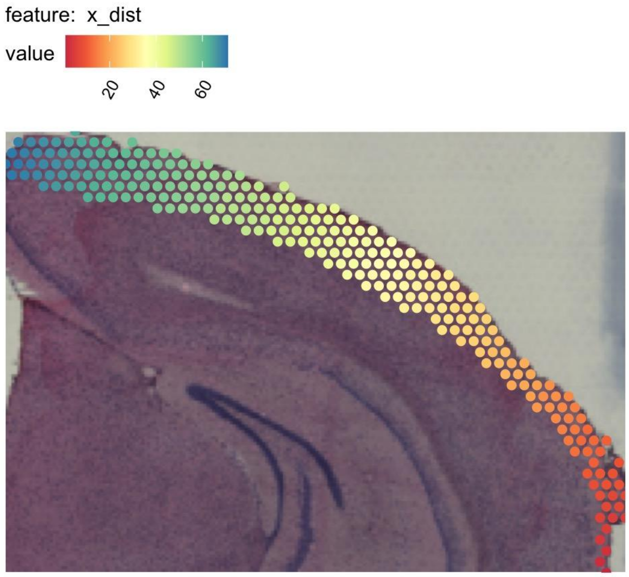

Supplementary Figure 14. *Distances between endpoints (spot colors) of a fully connected region (manually selected), calculated with AdjustTissueCoordinates.*

4. Comments on *semla* and other tools for SRT analysis and exploration

Spatial data format

The *semla* R package is developed as an updated and improved version of the previous *STUtility* R toolkit (Bergenstråhle et al. 2020), which utilizes the *Seurat* framework for storing expression data and adds an additional S4 object, termed “Staffli”, to hold the associated spatial data. This new version utilizes the Seurat framework for storing expression data and introduces an additional S4 object called "Staffli" to hold spatial data associated with it. If a user has already initialized a spatial object using Seurat's `Load10X_Spatial()` function, it can be converted to be compatible with *semla* using the `UpdateSeuratForSemla()` function. To facilitate visualization, specialized functions are provided in the *semla* package for plotting SRT data. These functions enable users to create images suitable for publication by offering features like image cropping, customizable scale bars, and extensive control over color scales and layouts. In the *semla* package, we have also included interactive web applications for smooth exploration and labeling of the user’s SRT data, and for aligning the data from multiple slices by applying image transformations such as rotation and scaling. To extract meaningful insights from the SRT data, *semla* further includes multiple functions to analyze the data and look for spatially relevant patterns.

The spatial omics field is rapidly evolving, with new computational tools and frameworks to handle the data being developed with high frequency. As of version 3.2 of Seurat (2020), they introduced new functionalities for processing Visium and Slide-seq data, allowing the user to visualize their spatial data and perform integration with annotated scRNA-seq data to infer spatial localization of cell types. With Seurat's latest release, the version 5 beta (2023), they have moreover added support for imaging based SRT data (Vizgen MERSCOPE, 10x Genomics Xenium, Nanostring CosMx, and Akoya CODEX) and further developed a few specialized methods such as niche identification. However, more specialized spatial analyses, such as those presented in the SquidPy and the Giotto packages, are still missing within Seurat. These spatial analyses may involve tools for computing spatial statistics or identification of spatial gene co-expression modules.

SpatialExperiment (Righelli et al. 2022) is another R based framework for efficient handling of SRT data, comparable to the AnnData format available in python. Toolkits utilizing the SpatialExperiment object format, such as spatialLIBD (Pardo et al. 2022), have started to emerge and provide a promising alternative avenue for SRT data analysis in R. Likely due to the youth of these toolkits, the size of the community of people developing new analysis methods is however limited for this object format. The same can moreover be said for R packages that have developed their own object structure for SRT data, such as Giotto (Dries et al. 2021) and SPATA2 (Kueckelhaus et al. 2023).

Interactive UI tools

Interactive visualization of SRT data is of great benefit for anyone interested in exploring their spatial data, both researchers with limited programming expertise and experienced bioinformaticians in need of browsing the data quickly and/or annotating selections of data points. 10x Genomics provides their Loupe Browser application for exploration of Visium data, which includes useful features such as spot annotation and rapid visualization of gene and cluster features, but is however limited to the use of the cloupe Space Ranger output files, one sample at the time. Seurat includes an interactive Shiny application for visualizing spatial data, though it is designed as a plain plotting tool for visualizing features and lacks any ability to create new spot labels. Other interactive UI applications for Visium data are available and address various aspects of interactive data exploration, such as multi-sample handling or incorporation of statistical analysis tools within the application. For instance, spatialLIBD is a powerful interactive UI application, powered by Shiny and Plotly, for exploring SRT data in a SpatialExperiment format. In comparison with the Feature Viewer provided in *semla*, it is not possible to return manual spot annotations to the R object in a one-step process with spatialLIBD, and it does moreover not handle

zooming of the tissue image in an efficient way that allows the user to utilize a high resolution image for detailed exploration of their data in its histological context.

Spatial platform compatibility

As mentioned previously, Seurat is currently able to handle SRT data generated from several platforms. The Giotto and Squidpy libraries also include support for additional packages than Visium. *Semla* on the other hand has been developed for the primary intent of processing and analyzing Visium Gene Expression data, where a matching histological image is available. Visium is to date the most widely used SRT platform, based on available published datasets, and the need for accessible analysis tools is therefore greater than for other platforms. Besides the original Visium Gene Expression platform for fresh frozen tissue samples, using the poly-A capture chemistry, *semla* can also handle output data from the probe-based Visium FFPE platform, with or without CytAssist (including the extra large (XL) capture areas), Visium with immunofluorescence (IF) images, and the under-development Visium high definition (HD) platform. For Visium IF, the images will have to be registered to the hematoxylin and eosin (H&E) stained section image, enabling the user to replace the H&E image with the IF image when loading the image with *semla*. However, the image processing functionalities provided in *semla*, such as masking of the background through tissue outline detection, have been optimized for H&E images and will not produce desirable results in its default mode. Instead, users can pass a custom method to the `MaskImages()` function in order to mask other types of images. The "custom masking" section in the "Mask images" tutorial provides some basic examples of how to create such a function (https://ludvigla.github.io/semla/articles/mask_images.html#custom-masking-advanced). Tissue detection is a non-trivial problem which makes it difficult to provide a universal technique and therefore users are encouraged to explore and build customized strategies using the *magick* R package.

Despite the Visium-centric development of *semla*, the package is however not necessarily limited to Visium data. As long as there is a feature*spot matrix and a spot coordinate file available, *semla* can import the data. Although, there are currently no functions within *semla* (v. 1.1) designed to handle output files in other formats than as generated by the 10x Space Ranger pipeline. Taking advantage of Seurat's spatial data support, there is nonetheless the possibility to import Slide-seq data into *semla* by converting the Slide-seq Seurat object into a *semla* compatible object, as outlined below and in the article "Slide-seq data" on the website (https://ludvigla.github.io/semla/articles/slide-seq.html). Given the lack of a histological image in Slide-seq data, all image related functionalities of *semla* will be inaccessible, including the Feature Viewer, when working with this data type. Other spatial analysis tools may

moreover work differently, and require some caution, given that most functions have been developed with the Visium data format in mind.

```
Unset
library(semla)
library(SeuratData)
InstallData("ssHippo")
slide_seq <- LoadData("ssHippo")
slide_seq_semla <- UpdateSeuratForSemla(slide_seq)
```

The part of the *semla* package allowing for additional spatially resolved omics platform support is still under development, and it is our ambition to make *semla* compatible with more platforms in order to allow for integrated multi-platform analyses in the future.

Conclusion

Our vision with a specialized R package for SRT data analysis, is that you should be able to perform all your desired exploratory work and spatial analyses with the convenience of not having to convert your R object for various analysis steps. Therefore, we see it as a large benefit to base *semla* on the widely popular Seurat object structure, which allows the users to tap into all tools developed for scRNA-seq analysis with Seurat and piggy-back on the fantastic development that the Seurat team produces with their latest version of the package. Initiating a new *semla* object with your Visium data, will thus automatically allow you to apply any Seurat compatible tool as well as open up the possibility to utilize all the specialized functions provided in *semla*. The framework of *semla* is designed to be flexible and allow for continuous development to include the potential support of SpatialExperiment data formats or support for additional spatial platforms besides Visium.

5. Limitations

Semla is an R package intended for processing, analysis, and visualization of SRT data, with a specific focus on the Visium Gene Expression platform. It is built to extend the Seurat toolbox and offers a wide variety of functions, designed with the intention to provide the basis for unleashing the full potential of your spatial data. While *semla* is readily available for use from its initial release, there are some limitations we would like to highlight.

SRT platform support. As mentioned, *semla* is currently designed specifically for Visium Gene Expression data. While this is the case, it is possible to load spatial data of other origins with *semla*, albeit limited in function compatibility of certain analysis tools and with a lack of easily applicable functions for loading other data types. These are aspects we aim to address with future updates of *semla*. In the meanwhile, we encourage users with other types of spatial omics data than Visium to use alternative packages, such as Seurat (v. 5 beta) or Giotto.

Data structure. *Semla* is built upon the Seurat framework, and relies on the Seurat object structure for storing expression assays, dimensionality reductions, meta data, and other associated data/information. For Visium datasets, *semla* adds additional information about the spot coordinates and optionally the H&E images. This additional information typically consumes much less memory compared to the count assays stored within the Seurat object, thus exerting minimal impact on performance. However, with Seurat v5, which at this time is a beta release, new infrastructure is offered to handle even larger datasets with millions of cells (or spots) even if the data cannot be fully loaded into memory. As of *selma's* current version (v. 1.1), we have not implemented support for any other format other than Seurat (e.g, SpatialExperiment), although *semla* has been designed to ease a future implementation of such support.

Image processing. While it is possible to load other images (PNG or JPEG format) than the H&E image provided among the Space Ranger output files, such as Visium IF images, it is important to ensure that its size and alignment is equivalent to that generated by the Space Ranger pipeline, which may require manual adjustment with an image editing software (e.g Adobe Photoshop). Image processing functions, such as `MaskImages()`, might fail for certain histological images, for instance when staining artifacts are present. There are a number of potentially useful image processing functionalities that are currently missing in *semla*, for instance cell segmentation tools or feature extraction methods.

Multi-sample alignment. *Semla* includes an interactive sample alignment tool which can be used for alignment of tissue sections with similar shape and histology, for instance consecutive tissue sections. The alignment tools handles rigid transformations such as rotation, scaling, and translation. Any non-linear global or local distortions of the samples would on the other hand be difficult to account for using only rigid transformations. Moreover, the alignment tool cannot perform spot-level alignment across multiple sections to form a common coordinate framework or a “consensus slice”. As this is a non-trivial task, we would recommend users looking to perform such transformations to explore other tools developed for this

particular purpose, for instance *eggplant* (Andersson et al. 2021) or *Probabilistic Alignment of ST Experiments* (PASTE) (Zeira et al. 2022).

6. References

Andersson, A., Bergenstråhle, J., Asp, M., Bergenstråhle, L., Jurek, A., Fernández Navarro, J., & Lundeberg, J. (2020). Single-cell and spatial transcriptomics enables probabilistic inference of cell type topography. Communications biology, 3(1), 565. https://doi.org/10.1038/s42003-020-01247-y

Andersson, A., Andrusivová, Z., Czarnewski, P., Li, X., Sundström, E., Lundeberg, J. (2021). A Landmark-based Common Coordinate Framework for Spatial Transcriptomics Data. bioRxiv 2021.11.11.468178; doi: https://doi.org/10.1101/2021.11.11.468178

Bergenstråhle, J., Larsson, L., & Lundeberg, J. (2020). Seamless integration of image and molecular analysis for spatial transcriptomics workflows. BMC genomics, 21(1), 482. https://doi.org/10.1186/s12864-020-06832-3

Bäckdahl, J., Franzén, L., Massier, L., Li, Q., Jalkanen, J., Gao, H., Andersson, A., Bhalla, N., Thorell, A., Rydén, M., Ståhl, P. L., & Mejhert, N. (2021). Spatial mapping reveals human adipocyte subpopulations with distinct sensitivities to insulin. Cell metabolism, 33(9), 1869–1882.e6. https://doi.org/10.1016/j.cmet.2021.07.018

Csárdi G, Salmon M (2023). rhub: Connect to 'R-hub'. https://github.com/r-hub/rhub, https://r-hub.github.io/rhub/.

Dries, R., Zhu, Q., Dong, R., Eng, C. L., Li, H., Liu, K., Fu, Y., Zhao, T., Sarkar, A., Bao, F., George, R. E., Pierson, N., Cai, L., & Yuan, G. C. (2021). Giotto: a toolbox for integrative analysis and visualization of spatial expression data. Genome biology, 22(1), 78. https://doi.org/10.1186/s13059-021-02286-2

Fernández Navarro, J., Lundeberg, J., & Ståhl, P. L. (2019). ST viewer: a tool for analysis and visualization of spatial transcriptomics datasets. Bioinformatics (Oxford, England), 35(6), 1058–1060. https://doi.org/10.1093/bioinformatics/bty714

Hao, Y., Hao, S., Andersen-Nissen, E., Mauck, W. M., 3rd, Zheng, S., Butler, A., Lee, M. J., Wilk, A. J., Darby, C., Zager, M., Hoffman, P., Stoeckius, M., Papalexi, E., Mimitou, E. P., Jain, J., Srivastava, A.,

Stuart, T., Fleming, L. M., Yeung, B., Rogers, A. J., … Satija, R. (2021). Integrated analysis of multimodal single-cell data. Cell, 184(13), 3573–3587.e29. https://doi.org/10.1016/j.cell.2021.04.048

Kleshchevnikov, V., Shmatko, A., Dann, E., Aivazidis, A., King, H. W., Li, T., Elmentaite, R., Lomakin, A., Kedlian, V., Gayoso, A., Jain, M. S., Park, J. S., Ramona, L., Tuck, E., Arutyunyan, A., Vento-Tormo, R., Gerstung, M., James, L., Stegle, O., & Bayraktar, O. A. (2022). Cell2location maps fine-grained cell types in spatial transcriptomics. Nature biotechnology, 40(5), 661–671. https://doi.org/10.1038/s41587-021-01139-4

Kueckelhaus J, Heiland D, Frerich S (2023). SPATA2: A Toolbox for Spatial Gene Expression Analysis. R package version 2.0.4, https://themilolab.com/.

Newman M. E. (2002). Assortative mixing in networks. Physical review letters, 89(20), 208701. https://doi.org/10.1103/PhysRevLett.89.208701

Parigi, S. M., Larsson, L., Das, S., Ramirez Flores, R. O., Frede, A., Tripathi, K. P., Diaz, O. E., Selin, K., Morales, R. A., Luo, X., Monasterio, G., Engblom, C., Gagliani, N., Saez-Rodriguez, J., Lundeberg, J., & Villablanca, E. J. (2022). The spatial transcriptomic landscape of the healing mouse intestine following damage. Nature communications, 13(1), 828. https://doi.org/10.1038/s41467-022-28497-0

Pardo, B., Spangler, A., Weber, L. M., Page, S. C., Hicks, S. C., Jaffe, A. E., Martinowich, K., Maynard, K. R., & Collado-Torres, L. (2022). spatialLIBD: an R/Bioconductor package to visualize spatially-resolved transcriptomics data. BMC genomics, 23(1), 434. https://doi.org/10.1186/s12864-022-08601-w

Righelli, D., Weber, L. M., Crowell, H. L., Pardo, B., Collado-Torres, L., Ghazanfar, S., Lun, A. T. L., Hicks, S. C., & Risso, D. (2022). SpatialExperiment: infrastructure for spatially-resolved transcriptomics data in R using Bioconductor. Bioinformatics (Oxford, England), 38(11), 3128–3131. https://doi.org/10.1093/bioinformatics/btac299

Ripley, B. D. (1976). The second-order analysis of stationary point processes. Journal of Applied Probability, 13(2), 255–266. Cambridge University Press.

Tabula Muris Consortium (2020). A single-cell transcriptomic atlas characterizes ageing tissues in the mouse. Nature, 583(7817), 590–595. https://doi.org/10.1038/s41586-020-2496-1

Tasic, B., Menon, V., Nguyen, T. N., Kim, T. K., Jarsky, T., Yao, Z., Levi, B., Gray, L. T., Sorensen, S. A., Dolbeare, T., Bertagnolli, D., Goldy, J., Shapovalova, N., Parry, S., Lee, C., Smith, K., Bernard, A.,

Madisen, L., Sunkin, S. M., Hawrylycz, M., … Zeng, H. (2016). Adult mouse cortical cell taxonomy revealed by single cell transcriptomics. Nature neuroscience, 19(2), 335–346. https://doi.org/10.1038/nn.4216

Zeira, R., Land, M., Strzalkowski, A., & Raphael, B. J. (2022). Alignment and integration of spatial transcriptomics data. Nature methods, 19(5), 567–575. https://doi.org/10.1038/s41592-022-01459-6
